# Supplementary material for: Emotions and Food Consumption: Emotional Eating Behavior in a European Population
Source: Foods. 2023 Feb 17;12(4):872. doi: 10.3390/foods12040872 (PMC9957014; doi:10.3390/foods12040872)
Supplement: Supplementary file 1 [file foods-12-00872-s001.zip › foods-2139193-supplementary.pdf]

Supplemental Table S1. Differences between study groups in emotional eating behavior during different emotional conditions (Post-Hoc Test p-values*; N = 9052)

	Cope with stress	Eating sweets when depressed	Consolation via eating when lonely	Boredom eating	Emotional consolation	Helping to control weight	Keeping awake and alert	Helping to relax	Making oneself feel good	Emotional eating behavior (overall)
Portugal vs. Croatia	0.001	<0.001	0.764	<0.001	<0.001	<0.001	<0.001	<0.001	<0.001	0.001
Portugal vs. Greece	<0.001	<0.001	<0.001	<0.001	<0.001	<0.001	<0.001	<0.001	0.805	<0.001
Portugal vs. Hungary	<0.001	0.013	0.001	0.017	0.302	<0.001	<0.001	<0.001	<0.001	<0.001
Portugal vs. Italy	<0.001	<0.001	<0.001	<0.001	<0.001	<0.001	<0.001	<0.001	0.990	<0.001
Portugal vs. Latvia	<0.001	<0.001	<0.001	<0.001	<0.001	<0.001	<0.001	<0.001	<0.001	<0.001
Portugal vs. Lithuania	<0.001	<0.001	<0.001	<0.001	<0.001	<0.001	<0.001	<0.001	0.006	<0.001
Portugal vs. Poland	<0.001	<0.001	<0.001	<0.001	<0.001	<0.001	<0.001	<0.001	0.003	<0.001
Portugal vs. Romania	<0.001	<0.001	<0.001	<0.001	0.309	<0.001	<0.001	<0.001	<0.001	<0.001
Portugal vs. Slovenia	<0.001	<0.001	<0.001	<0.001	0.045	<0.001	<0.001	<0.001	<0.001	0.002
Portugal vs. The Netherland	<0.001	<0.001	<0.001	<0.001	<0.001	<0.001	<0.001	<0.001	<0.001	<0.001
Portugal vs. Serbia	0.018	0.482	0.018	<0.001	<0.001	<0.001	<0.001	<0.001	<0.001	0.001
Croatia vs. Greece	<0.001	0.002	<0.001	<0.001	0.112	0.599	0.005	0.020	<0.001	0.001
Croatia vs. Hungary	<0.001	0.123	0.002	<0.001	<0.001	<0.001	<0.001	<0.001	<0.001	<0.001
Croatia vs. Italy	<0.001	<0.001	<0.001	<0.001	0.578	0.096	0.213	0.035	<0.001	<0.001
Croatia vs. Latvia	<0.001	<0.001	<0.001	<0.001	<0.001	<0.001	0.004	<0.001	<0.001	<0.001
Croatia vs. Lithuania	<0.001	<0.001	<0.001	<0.001	<0.001	<0.001	0.004	<0.001	<0.001	<0.001
Croatia vs. Poland	<0.001	<0.001	<0.001	<0.001	0.549	<0.001	0.667	<0.001	0.020	<0.001
Croatia vs. Romania	<0.001	<0.001	<0.001	0.194	<0.001	<0.001	<0.001	<0.001	<0.001	<0.001
Croatia vs. Slovenia	<0.001	<0.001	<0.001	0.497	<0.001	<0.001	<0.001	<0.001	0.005	0.299
Croatia vs. The Netherland	<0.001	<0.001	<0.001	<0.001	0.866	<0.001	<0.001	<0.001	0.697	0.111
Croatia vs. Serbia	0.978	0.002	0.036	0.933	<0.001	<0.001	<0.001	0.001	<0.001	<0.001
Greece vs. Hungary	<0.001	0.001	<0.001	<0.001	<0.001	<0.001	<0.001	<0.001	<0.001	<0.001
Greece vs. Italy	0.461	0.335	0.998	0.824	0.385	0.376	0.144	0.770	0.906	0.598

Greece vs. Latvia	0.154	0.664	<0.001	0.005	<0.001	0.090	<0.001	0.097	<0.001	<0.001
Greece vs. Lithuania	<0.001	0.001	<0.001	<0.001	<0.001	0.072	<0.001	0.051	0.130	<0.001
Greece vs. Poland	0.667	0.597	0.040	0.979	0.353	<0.001	0.029	0.001	0.056	0.362
Greece vs. Romania	0.515	0.905	0.997	0.009	0.028	<0.001	<0.001	0.528	<0.001	0.614
Greece vs. Slovenia	0.485	0.558	0.061	0.001	0.052	<0.001	0.666	<0.001	<0.001	0.018
Greece vs. The Netherland	0.723	0.754	0.075	0.014	0.133	<0.001	0.379	<0.001	<0.001	0.138
Greece vs. Serbia	0.006	<0.001	<0.001	0.002	<0.001	<0.001	<0.001	0.335	<0.001	<0.001
Hungary vs. Italy	<0.001	<0.001	<0.001	<0.001	<0.001	0.001	<0.001	<0.001	<0.001	<0.001
Hungary vs. Latvia	<0.001	<0.001	<0.001	<0.001	<0.001	0.005	0.070	<0.001	<0.001	<0.001
Hungary vs. Lithuania	<0.001	<0.001	<0.001	<0.001	<0.001	0.013	0.873	<0.001	<0.001	<0.001
Hungary vs. Poland	<0.001	0.001	<0.001	<0.001	<0.001	<0.001	<0.001	<0.001	<0.001	<0.001
Hungary vs. Romania	<0.001	<0.001	<0.001	<0.001	0.073	<0.001	0.044	<0.001	0.155	<0.001
Hungary vs. Slovenia	<0.001	<0.001	<0.001	<0.001	0.017	<0.001	<0.001	0.320	0.038	<0.001
Hungary vs. The Netherland	<0.001	<0.001	<0.001	<0.001	<0.001	0.816	<0.001	0.965	<0.001	<0.001
Hungary vs. Serbia	<0.001	0.259	0.362	<0.001	0.001	0.021	0.482	<0.001	0.503	0.025
Italy vs. Latvia	0.451	0.461	<0.001	0.005	<0.001	0.381	<0.001	0.036	<0.001	<0.001
Italy vs. Lithuania	0.001	0.026	<0.001	<0.001	<0.001	0.315	<0.001	0.019	0.056	<0.001
Italy vs. Poland	0.203	0.101	0.023	0.815	0.999	<0.001	0.437	<0.001	0.044	0.711
Italy vs. Romania	0.122	0.213	0.963	0.002	0.001	<0.001	<0.001	0.278	<0.001	0.946
Italy vs. Slovenia	0.875	0.571	0.040	<0.001	0.002	<0.001	0.027	<0.001	<0.001	0.002
Italy vs. The Netherland	0.238	0.444	0.053	0.019	0.524	<0.001	0.012	<0.001	<0.001	0.033
Italy vs. Serbia	<0.001	<0.001	<0.001	<0.001	<0.001	<0.001	<0.001	0.199	<0.001	<0.001
Latvia vs. Lithuania	0.010	<0.001	0.001	0.221	0.004	0.863	0.023	0.641	<0.001	0.091
Latvia vs. Poland	0.033	0.267	<0.001	0.001	<0.001	<0.001	<0.001	0.047	<0.001	<0.001
Latvia vs. Romania	0.015	0.680	<0.001	<0.001	<0.001	<0.001	0.731	0.233	<0.001	<0.001
Latvia vs. Slovenia	0.285	0.714	<0.001	<0.001	<0.001	<0.001	<0.001	<0.001	<0.001	<0.001
Latvia vs. The Netherland	0.043	0.927	0.034	0.692	<0.001	0.001	<0.001	<0.001	<0.001	<0.001

Latvia vs. Serbia	<0.001	<0.001	<0.001	<0.001	<0.001	<0.001	0.005	0.545	<0.001	<0.001
Lithuania vs. Poland	<0.001	<0.001	<0.001	<0.001	<0.001	<0.001	<0.001	0.283	<0.001	<0.001
Lithuania vs. Romania	<0.001	<0.001	<0.001	<0.001	<0.001	<0.001	0.017	0.070	<0.001	<0.001
Lithuania vs. Slovenia	<0.001	0.002	<0.001	<0.001	<0.001	<0.001	<0.001	<0.001	<0.001	<0.001
Lithuania vs. The Netherland	<0.001	0.002	<0.001	0.107	<0.001	0.003	<0.001	<0.001	<0.001	<0.001
Lithuania vs. Serbia	<0.001	<0.001	<0.001	<0.001	<0.001	<0.001	0.562	0.339	<0.001	<0.001
Poland vs. Romania	0.756	0.602	0.036	0.002	<0.001	0.620	<0.001	<0.001	<0.001	0.651
Poland vs. Slovenia	0.224	0.177	0.719	<0.001	0.001	0.092	0.002	<0.001	<0.001	<0.001
Poland vs. The Netherland	0.996	0.333	<0.001	0.005	0.486	<0.001	0.001	<0.001	0.026	0.008
Poland vs. Serbia	0.005	<0.001	<0.001	0.001	<0.001	<0.001	0.001	0.040	<0.001	<0.001
Romania vs. Slovenia	0.107	0.387	0.034	0.518	0.545	0.217	<0.001	<0.001	0.409	0.002
Romania vs. The Netherland	0.748	0.660	0.057	<0.001	<0.001	<0.001	<0.001	<0.001	0.016	<0.001
Romania vs. Serbia	0.017	<0.001	<0.001	0.410	<0.001	<0.001	0.004	0.515	0.046	<0.001
Slovenia vs. The Netherland	0.263	0.747	<0.001	<0.001	<0.001	<0.001	0.538	0.347	0.095	0.481
Slovenia vs. Serbia	<0.001	<0.001	<0.001	0.639	<0.001	<0.001	<0.001	<0.001	0.007	<0.001
The Netherland vs. Serbia	0.009	<0.001	<0.001	<0.001	<0.001	0.023	<0.001	<0.001	<0.001	<0.001

Note: *Mann Whintey U test; Croatia (N = 1538); Greece (N = 498); Hungary (N = 500); Italy (N = 541); Latvia (N = 636); Lithuania (N= 507); The Netherlands (N = 521); Poland (N = 586); Portugal (N = 1314); Romania (N = 821); Serbia (N = 498); Slovenia (N = 1093).

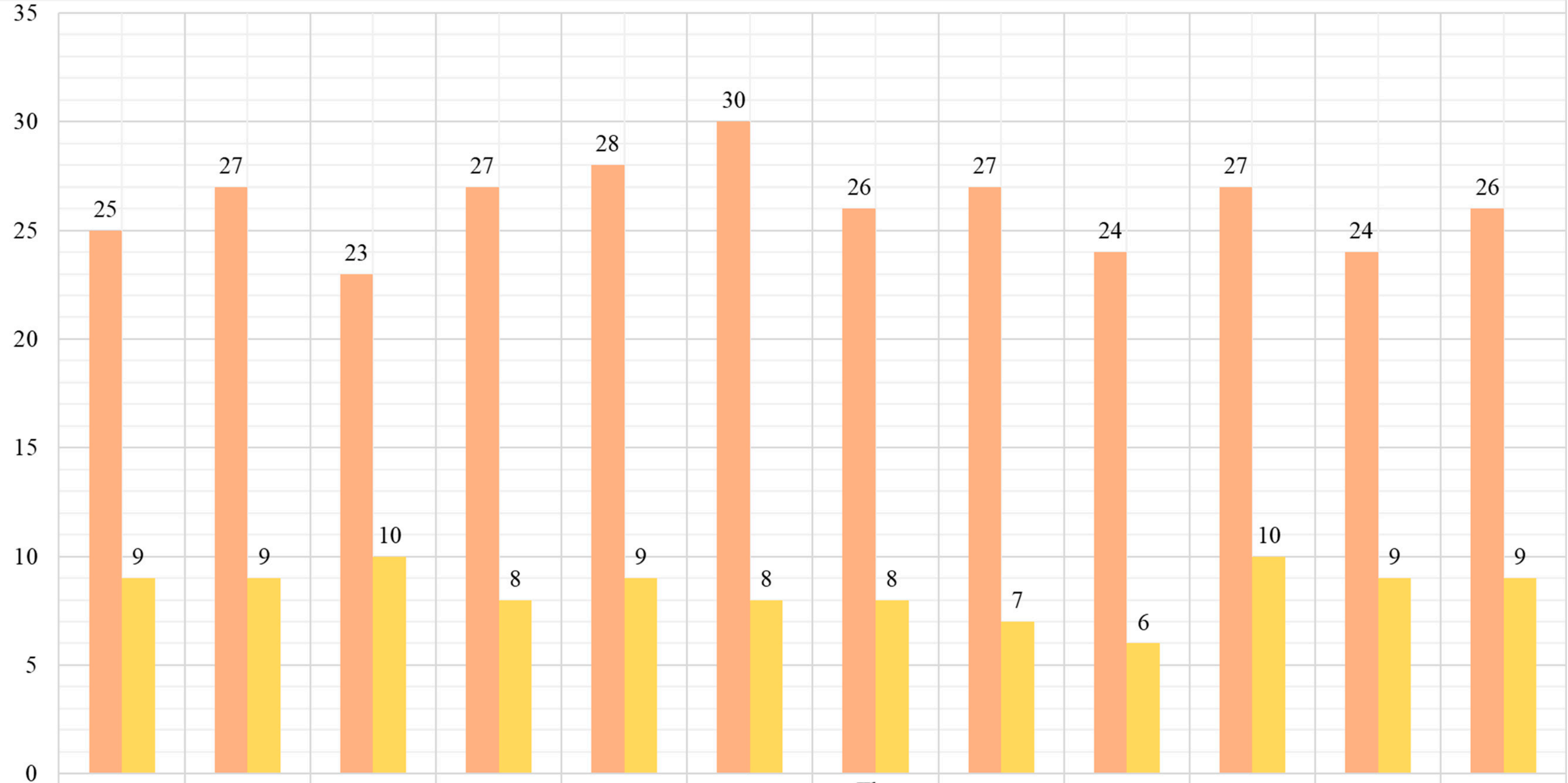

	Croatia	Greece	Hungary	Italy	Latvia	Lithuania	The Netherlands	Poland	Portugal	Romania	Serbia	Slovenia
Median	25	27	23	27	28	30	26	27	24	27	24	26
Interquartile range	9	9	10	8	9	8	8	7	6	10	9	9

Supplemental Figure S1. Distribution of the emotional eating behavior in a European study population (ranging from 9 to 45).

Motivations for Food Choices Questionnaire: EATMOT

This questionnaire aims to collect data to conduct a research on the factors that influence food choice in different countries, integrated in an international project lead by CI&DETS Research Centre of the Polytechnic Institute of Viseu, Portugal (PROJ/CI&DETS/2016/0008: EATMOT).

The participation is voluntary and all information collected is strictly confidential.

Thank you in advance for your cooperation, without which it would not be possible to realize this project. Remember that there are no good or bad answers, do not hesitate to respond.

Reserved to the interviewer

Number of questionnaire			
Date		Place of interview	

Target

General public	Yes ☐ 1	No ☐ 2 Target group:____________________(2a)

I. DEMOGRAPHICS DATA

The requested demographic data serves only to interpret the responses, and are absolutely confidential.

(Fill out by inserting an X or completing the spaces available).

1. **Age: _________** years

2. **Gender:**

Female ☐1 Male ☐2

3. **Highest education level completed:**

Primary school ☐ 1 Secondary school ☐ 2 University ☐ 3

4. **Country of residence ______________________**

5. **Living environment:**

Rural ☐ 1 Urban ☐ 2 Suburban ☐ 3

6. **Civil state:**

Single ☐ 1 Married/Living together ☐ 2 Divorced/Separated ☐ 3 Widow ☐ 4

7. **Present professional activity:**

Employed ☐ 1 Unemployed ☐ 2 Student ☐ 3

Retired ☐ 4 Student worker ☐ 5

8. **Professional activity /studies related to any of the following areas:**

Nutrition ☐ 1 Food ☐ 2 Agriculture ☐ 3 Sports ☐ 4 Psychology ☐ 5

Health related activities ☐ 6 Which? ______________________________ (6.a)

The professional activity is not related to any of the above areas ☐ 7

9. **Are you responsible for buying the food you eat?**

Yes ☐ 1 No ☐ 2

II. ANTHROPOMETRIC DATA AND BEHAVIOURAL & HEALTH RELATED ELEMENTS

To the best of your knowledge please fill in your current weight and height:

10. **Height: ___________** meters or ___________ inches

11. **Weight: ___________** kg or ___________ pounds

12. **How often do you usually engage in physical exercise?**

Never ☐ 1 Sporadically (less than once/week) ☐ 2 Occasionally (once/week) ☐ 3

Moderately (2-3 times/week) ☐ 4 Intensively (+3 times/week) ☐ 5

13. **On average, how many hours per day do you spend watching TV or in front of the computer? ___________ hours**

14. **How often do you think that you follow a balanced/healthy diet?**

Never ☐ 1 Rarely ☐ 2 Sometimes ☐ 3 Frequently ☐ 4 Always ☐ 5

15. **Do you practice any specific voluntary dietary regimen?**

Raw foodism *(dietary practice of eating only uncooked, unprocessed foods)* ☐ 1

Fruitarianism *(diet that consists entirely or primarily of fruits and possibly nuts and seeds, without any animal products)* ☐ 2

Vegetarianism *(exclusion of any meat and may also include exclusion from by-products of animal slaughter)* ☐ 3

Veganism *(exclusion from animal products, and an associated philosophy that rejects the commodity status of animals)* ☐ 4

Flexitarianism *(plant-based diet with the occasional inclusion of animal products)* ☐ 5

Caloric restriction/Weight control ☐ 6

Religious restrictions ☐ 7 Which? ______________________________ (7.a)

Other ☐ 8 Which? ______________________________ (8.a)

I practice a general food regimen ☐ 9

16. Do you have any chronic disease?

Cardiovascular diseases ☐ 1 Diabetes ☐ 2 High cholesterol ☐ 3

Arterial hypertension ☐ 4 Gastric disorders ☐ 5 Intestinal disorders ☐ 6

Obesity ☐ 7 Other ☐ 8 Which? ______________________________ (8.a)

I do not suffer from chronic diseases ☐ 9

17. Do you have any food allergie/intolerance?

Lactose intolerance ☐ 1 Casein ☐ 2 Gluten ☐ 3 Nuts ☐ 4 Shellfish ☐ 5

Other ☐ 6 Which? ______________________________ (6.a)

I do not suffer from food allergies/intolerances ☐ 7

18. Did you at any time of your life experience an episode of any eating disorders?

Bulimia ☐ 1 Anorexia ☐ 2 Binge-eating ☐ 3 Other ☐ 4 Which? ______________________ (4.a)

I never experienced any eating disorders ☐ 5

III. PERCEPTIONS ABOUT A HEALTHY EATING

Indicate, on the scale shown between Strongly disagree and Strongly agree, your opinion about the following statements.

(Fill out by inserting an X).

	Strongly disagree	Disagree	Neither agree nor disagree	Agree	Strongly agree
19.1 A healthy diet is based on calorie count	☐ 1	☐ 2	☐ 3	☐ 4	☐ 5

19.2 We should never consume sugary products	☐ 1	☐ 2	☐ 3	☐ 4	☐ 5
19.3 Fruit and vegetables are very important to a practice of a healthy eating	☐ 1	☐ 2	☐ 3	☐ 4	☐ 5
19.4 A healthy diet should be balanced, varied and complete	☐ 1	☐ 2	☐ 3	☐ 4	☐ 5
19.5 We can eat everything, as long as it is in small quantities	☐ 1	☐ 2	☐ 3	☐ 4	☐ 5
19.6 I believe that a healthy diet is not cheap	☐ 1	☐ 2	☐ 3	☐ 4	☐ 5
19.7 In my opinion it is strange that some people have cravings for sweets	☐ 1	☐ 2	☐ 3	☐ 4	☐ 5
19.8 I believe that tradition is very important to a healthy diet	☐ 1	☐ 2	☐ 3	☐ 4	☐ 5
19.9 I believe that food produced in a biological way is healthier	☐ 1	☐ 2	☐ 3	☐ 4	☐ 5
19.10 We should never consume fat products	☐ 1	☐ 2	☐ 3	☐ 4	☐ 5

IV. SOURCES OF INFORMATION ABOUT HEALTHY DIET

Indicate, on the scale shown between Never and Always, with what frequency you find information about eating a healthy diet.

(Fill out by inserting an **X**).

20. Where do you usually find information about eating a healthy diet?

	Never	Sporadically	Sometimes	Frequently	Always
20.1 Health centres, hospitals, general practitioner	☐ 1	☐ 2	☐ 3	☐ 4	☐ 5
20.2 Radio	☐ 1	☐ 2	☐ 3	☐ 4	☐ 5
20.3 Television	☐ 1	☐ 2	☐ 3	☐ 4	☐ 5

20.4 School	☐ 1	☐ 2	☐ 3	☐ 4	☐ 5
20.5 Magazines, books, newspapers	☐ 1	☐ 2	☐ 3	☐ 4	☐ 5
20.6 Internet	☐ 1	☐ 2	☐ 3	☐ 4	☐ 5
20.7 Family, friends	☐ 1	☐ 2	☐ 3	☐ 4	☐ 5

V. HEALTHY MOTIVATIONS

Indicate, on the scale shown between Strongly disagree and Strongly agree, your opinion about the following statements.

(Fill out by inserting an X).

	Strongly disagree	Disagree	Neither agree nor disagree	Agree	Strongly agree
21.1 I am very concerned about the hygiene and safety of the food I eat	☐ 1	☐ 2	☐ 3	☐ 4	☐ 5
21.2 It is important for me that my diet is low in fat	☐ 1	☐ 2	☐ 3	☐ 4	☐ 5
21.3 Usually I follow a healthy and balanced diet	☐ 1	☐ 2	☐ 3	☐ 4	☐ 5
21.4 It is important for me that my daily diet contains a lot of vitamins and minerals	☐ 1	☐ 2	☐ 3	☐ 4	☐ 5
21.5 There are some foods that I consume regularly, even if they may raise my cholesterol	☐ 1	☐ 2	☐ 3	☐ 4	☐ 5
21.6 I try to eat foods that do not contain additives	☐ 1	☐ 2	☐ 3	☐ 4	☐ 5
21.7 I avoid eating processed foods, because of their lower nutritional quality	☐ 1	☐ 2	☐ 3	☐ 4	☐ 5
21.8 It is important for me to eat food that keeps me healthy	☐ 1	☐ 2	☐ 3	☐ 4	☐ 5

21.9 There are some foods that I consume regularly, even if they may raise my blood glycaemia	☐ 1	☐ 2	☐ 3	☐ 4	☐ 5
21.10 I avoid foods with genetically modified organisms	☐ 1	☐ 2	☐ 3	☐ 4	☐ 5

VI. EMOTIONAL MOTIVATIONS

Indicate, on the scale shown between Strongly disagree and Strongly agree, your opinion about the following statements.

(Fill out by inserting an X).

	Strongly disagree	Disagree	Neither agree nor disagree	Agree	Strongly agree
22.1 Food helps me cope with stress	☐ 1	☐ 2	☐ 3	☐ 4	☐ 5
22.2 I usually eat food that helps me control my weight	☐ 1	☐ 2	☐ 3	☐ 4	☐ 5
22.3 I often consume foods that keep me awake and alert (such as coffee, coke, energy drinks)	☐ 1	☐ 2	☐ 3	☐ 4	☐ 5
22.4 I often consume foods that helps me relax (such as some teas, red wine)	☐ 1	☐ 2	☐ 3	☐ 4	☐ 5
22.5 Food makes me feel good	☐ 1	☐ 2	☐ 3	☐ 4	☐ 5
22.6 When I feel lonely, I console myself by eating	☐ 1	☐ 2	☐ 3	☐ 4	☐ 5
22.7 I eat more when I have nothing to do	☐ 1	☐ 2	☐ 3	☐ 4	☐ 5
22.8 For me, food serves as an emotional consolation	☐ 1	☐ 2	☐ 3	☐ 4	☐ 5
22.9 I have more cravings for sweets when I am depressed	☐ 1	☐ 2	☐ 3	☐ 4	☐ 5

Thank you for you precious collaboration!
